# Use of period analysis to provide a timely assessment of 5-year relative survival for pancreatic cancer patients from Taizhou, eastern China

**DOI:** 10.1186/s12885-023-11119-3

**Published:** 2023-07-10

**Authors:** Ye Lu, Min He, Liyou Lian, Huijun Lei, Yongran Cheng, Liangyou Wang, Tianhui Chen, Jinfei Chen

**Affiliations:** 1 Department of Cancer Prevention, Zhejiang Cancer Hospital, 310022 Hangzhou, China; 2 Hangzhou Institute of Medicine (HIM), Chinese Academy of Sciences, Hangzhou, 310018 China; 3 Department of Oncology, the First Affiliated Hospital of Wenzhou Medical University, Wenzhou, 325015 China; 4 School of Public Health, Hangzhou Medical College, Hangzhou, 311300 China; 5 Department of Non-communicable Chronic Disease Control and Prevention, Taizhou Municipal Center for Disease Control and Prevention, Taizhou, 318000 China; 6 Department of Preventative Medicine, School of Medicine, Ningbo University, Ningbo, 315211 China

**Keywords:** Pancreatic cancer, Period analysis, 5-year relative survival, Cancer registration, Eastern China

## Abstract

Assessing long-term tumor survival rates is crucial for evaluating the effectiveness of tumor treatment and burden. However, timely assessment of long-term survival in patients with pancreatic cancer is lagging in China. In this study, we applied period analysis to estimate the long-term survival of pancreatic cancer patients using data from four population-based cancer registries in Taizhou city, eastern China. A total of 1121 patients diagnosed with pancreatic cancer between 2004 and 2018 were included. We assessed the 5-year relative survival (RS) using period analysis and further stratified by sex, age at diagnosis, and region. The 5-year RS during 2014–2018 overall reached 18.9% (14.7% for men and 23.3% for women, respectively). A decrease of the 5-year RS from 30.3% to 11.2% was observed in four diagnostic age gradients (< 55, 55–64, 65–74, and > 74 years age groups). The 5-year RS was higher in urban (24.2%) than in rural (17.4%) areas. Moreover, the 5-year RS of pancreatic cancer patients showed an overall increasing trend for the three periods (2004–2008, 2009–2013, and 2014–2018). Our study, using period analysis for the first time in China, provides the latest estimates of the survival of patients with pancreatic cancer, which provides essential evidence for the prevention and intervention of pancreatic cancer. The results also indicate the importance of further applications of the period analysis for more up-to-date and accurate survival estimates.

**Supplementary Information:**

The online version contains supplementary material available at 10.1186/s12885-023-11119-3.

## Background

Pancreatic cancer is one of the most lethal cancers and ranks the seventh leading cause of cancer-related deaths. About 495,773 new cases and 466,003 new deaths due to pancreatic cancer occurred in 2020 globally, with the mortality rate closely approaching the incidence rate [1]. Both the incidence and mortality rates for pancreatic cancer have increased worldwide [2]. Meanwhile, the rates vary widely by country, with the highest rates in the European Union and much lower rates among less developed countries in Africa and South Asia, showing a difference in the disease burden of pancreatic cancer in developed countries compared to developing countries [3, 4]. China is undergoing a change in the cancer spectrum from a developing country to a developed country, with a rapidly increased cancer burden, including pancreatic cancer [5]. In China, the latest estimated number of new cases and deaths due to pancreatic cancer in 2016 was 100,400 and 87,900, ranking the 10th and 6th for incidence and mortality rates among all malignant tumors, respectively [6].

It is essential to timely assess the long-term survival of cancer patients for clinical management and public health interventions, which are necessary to reduce the burden and improve the survival of cancers. The 5-year survival rate is the most used index for the evaluation of tumor burden. Generally, the observed survival rate obtained by a follow-up to 5 years and calculating the proportion of the number of people who lived for five years to the total number of people diagnosed with tumors in the period of interest is the truest and most accurate, but it is too lagging. To obtain a timely prognosis for patients diagnosed with cancer, the survival analysis method is generally used to estimate the 5-year relative survival (RS) rate. However, traditional survival analysis methods such as the cohort and complete method incorporate survival information before the period of interest, which has to use follow-up data of years, have apparent lag. They cannot accurately reflect the latest survival of cancer patients. The period analysis method proposed by Brenner has more advantages in terms of timeliness and accuracy of survival analysis [7–9]. The period analysis method includes all cases within the period of interest, which does not require follow-up data, calculating the actual survival estimates of newly diagnosed patients. It has been proved that period analysis provided more precise cancer survival estimates than cohort and complete methods and was widely used in the western [10–14]. However, in China, the application of the period approach has been scarce.

Our group has recently used the period analysis in the Chinese population [15–19]. We systematically compared the performance of cohort, complete, and period analysis using cancer registry data between 2009 and 2013 from Taizhou, eastern China. The findings supported that the period analysis provided more up-to-date, precise estimates of long-term survival overall and the stratification by sex, age at diagnosis, region, and cancer types, compared to the other two methods [15].

While timely assessment of long-term survival for patients with pancreatic cancer is essential for evaluating early detection and screening programs for pancreatic cancer, those data are incredibly scarce in China. Thereby, using period analysis, we aimed to provide the most up-to-date (during 2014–2018) estimates of 5-year RS for pancreatic cancer patients from Taizhou, eastern China, for overall and the stratification by sex, age at diagnosis, and region. We also aimed to assess the trends of 5-year RS during 2004–2018.

## Methods

### Data source

Taizhou City, located on the eastern coast of China, has a population of approximately 10% of Zhejiang Province. Data for incident cancer cases were obtained from the Information and Management System for Zhejiang Provincial Chronic Disease Surveillance, which is a platform established in 2001 for monitoring the incidence and mortality rates of chronic diseases (including cancer) in the region. Nine cancer registries in Taizhou provided data for this study, and data quality was assessed based on the proportion of death certification only (DCO) cases among all registered cancer cases. Registries with an overall proportion of DCO cases less than 13% from 2004 to 2018 were selected for further analysis, following a criterion recommended by Brenner for good quality cancer registries. Data from four counties (Luqiao, Yuhuan, Xianju, and Wenling, each with an overall proportion of DCO cases ranging from 9.8 to 12.1%) met this criterion and were included in the study.

Pancreatic cancer cases were identified using the tenth edition of the International Classification of Diseases (ICD-10) with code C25.0-C25.9. A total of 1751 cases diagnosed between 2001 and 2018 and followed up by December 31, 2018, were extracted from the database. We excluded 147 cases without clear diagnosis information, 293 cases lost to follow-up (16.7% of the initially identified cases, due to moving out of the area or transferring care to another hospital), and 190 with logical variable inconsistencies. Finally, 1121 cases were included in the survival analysis.

### Statistical analysis

The 5-year RS for pancreatic cancer patients was calculated as the ratio of observed survival in patients divided by the expected survival from a comparable group from the general population [20]. The expected survival was derived using the Ederer II method, based on the life table for the four counties of Taizhou (Luqiao, Yuhuan, Xianju, and Wenling) [15].

Period analysis was used to calculate the 5-year RS of pancreatic cancer patients for 2014–2018, with further stratification by sex, age at diagnosis, and region. Patients newly diagnosed during the interest period (2014–2018) and diagnosed before the interest period (2009–2013) and still surviving within the interest period were included in the study. The follow-up period was 2014–2018. The formula has been described in detail elsewhere [16, 19] (Supplementary Material).

The 5-year RS of pancreatic cancer patients for the 2004–2008 and 2009–2013 periods were also calculated using the period analysis method. Table 1 summarized data used for survival estimates of three periods. We further described the survival trends for patients by the established generalized linear model (GLM). The fitted models allowed to assess changes in survival trends by sex, age at diagnosis, or region.

**Table 1:**
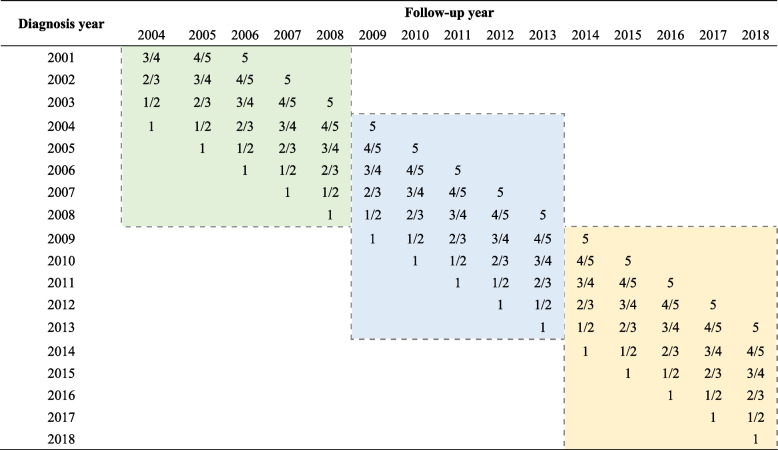
Illustration of data used to calculate the 5-year relative survival estimates for patients diagnosed in 2004–2008, 2009–2013, and 2014–2018 periods by period analysis

The colored areas indicate the data to calculate the 5-year relative survival estimates for three periods respectively. The numbers within the cells indicate the years of follow-up since diagnosis

All statistical analyses were performed by R version 3.13 (R Foundation for Statistical Computing, Vienna, Austria) using the function of GLM and the package ‘periodR’ [21].

## Results

### Basic characteristics of the patients

The characteristics of patients diagnosed with pancreatic cancer from January 2004 to December 2018 in 4 counties of Taizhou City, eastern China, are shown in Table 2. A total of 1121 cases were included in this analysis, containing 896 males and 225 females. The average age at diagnosis of patients was 70 years. The number of patients diagnosed at the age of < 55, 55–64, 65–74, and > 74 years old were 134, 230, 345, and 412 cases, respectively, while the age group of > 74 years accounted for the most significant proportion. Moreover, the number of patients living in rural areas (*n* = 989) was much larger than in urban areas (*n* = 132).

**Table 2:** Characteristics of pancreatic cancer patients diagnosed during 2004–2018 in Taizhou, eastern China

Characteristics	Number of cases	Diagnosed interval		
		2004-2008	2009-2013	2014-2018
Total	1121	107	301	713
Sex				
Male	896	82	286	528
Female	225	25	15	185
Region				
Urban area	132	22	45	65
Rural area	989	85	256	648
Average age (years)	70	72.4	69.6	68.3
Age at diagnosis (years)				
<55	134	14	37	83
55-64	230	22	52	156
65-74	345	39	93	213
＞74	412	32	119	261

### Five-year relative survival of pancreatic cancer

As shown in Table 3, the estimated 5-year RS of pancreatic cancer patients was 18.9% in the 2014–2018 interval, with estimates of 14.7% and 23.3% for males and females, respectively. With the increase in diagnosed age, the estimated 5-year RS was decreasing (30.3%, 23.2%, 15.9%, and 11.2% for patients diagnosed at age < 55, 55–64, 65–74, and > 74 years old, respectively), showing a solid age gradient. Besides, the 5-year RS of patients in the urban area (24.2%) was higher than that in the rural area (17.4%).

**Table 3:** The 5-year relative survival of pancreatic cancer patients during 2014 to 2018 in Taizhou, eastern China

	Estimated value (%)	Standard error(SE)
Total	18.9	1.9
Sex		
Male	14.7	2.3
Female	23.3	3.2
Age at diagnosis (years)		
< 55	30.3	3.0
55–64	23.2	3.1
65–74	15.9	3.1
> 74	11.2	3.6
Region		
Urban area	24.2	4.6
Rural area	17.4	2.3

**Fig. 1:**
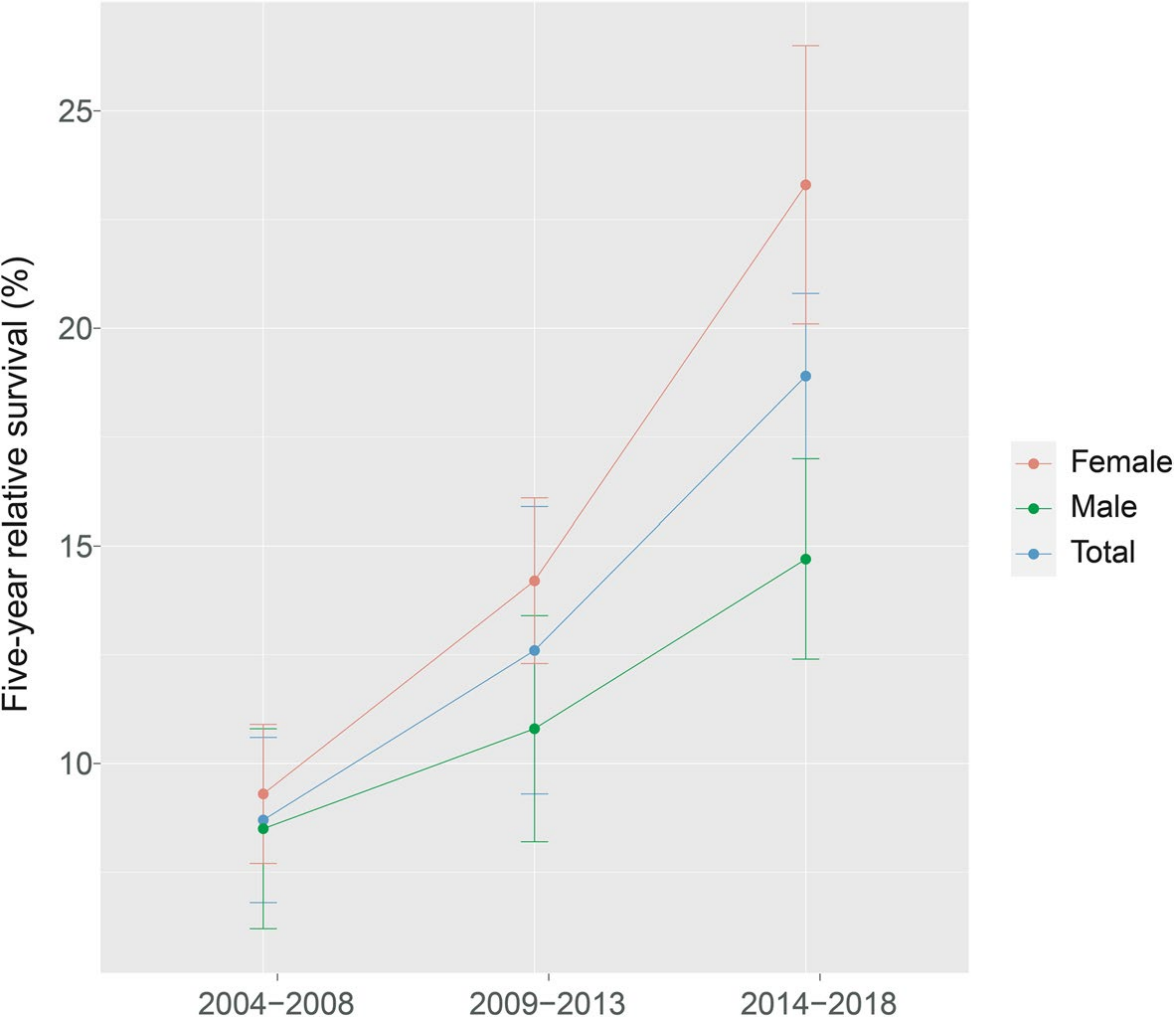
Five-year relative survival for total, male and female pancreatic cancers in 2004–2008, 2009–2013, and 2014–2018

**Fig. 2:**
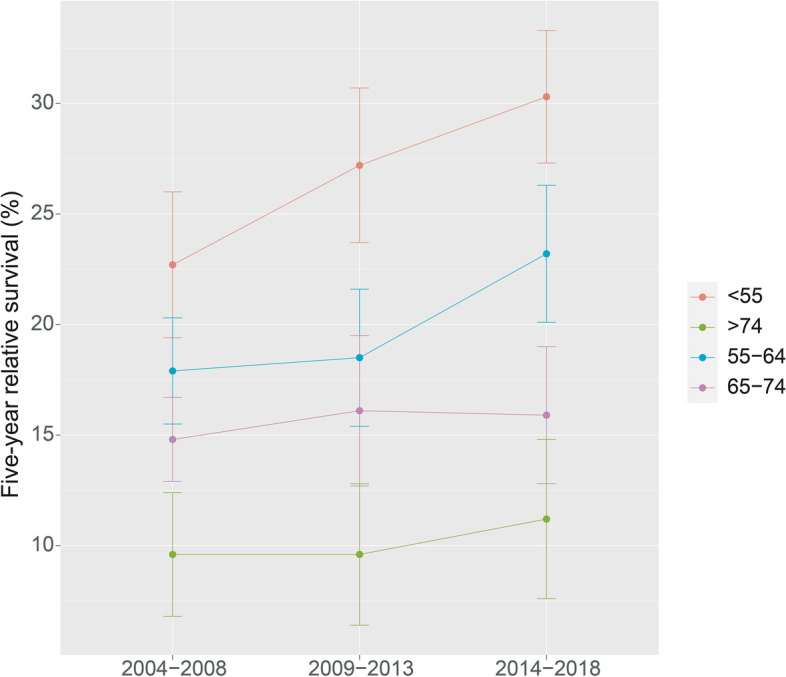
Five-year relative survival of pancreatic cancers for different ages at diagnosis in 2004–2008, 2009–2013, and 2014–2018

**Fig. 3:**
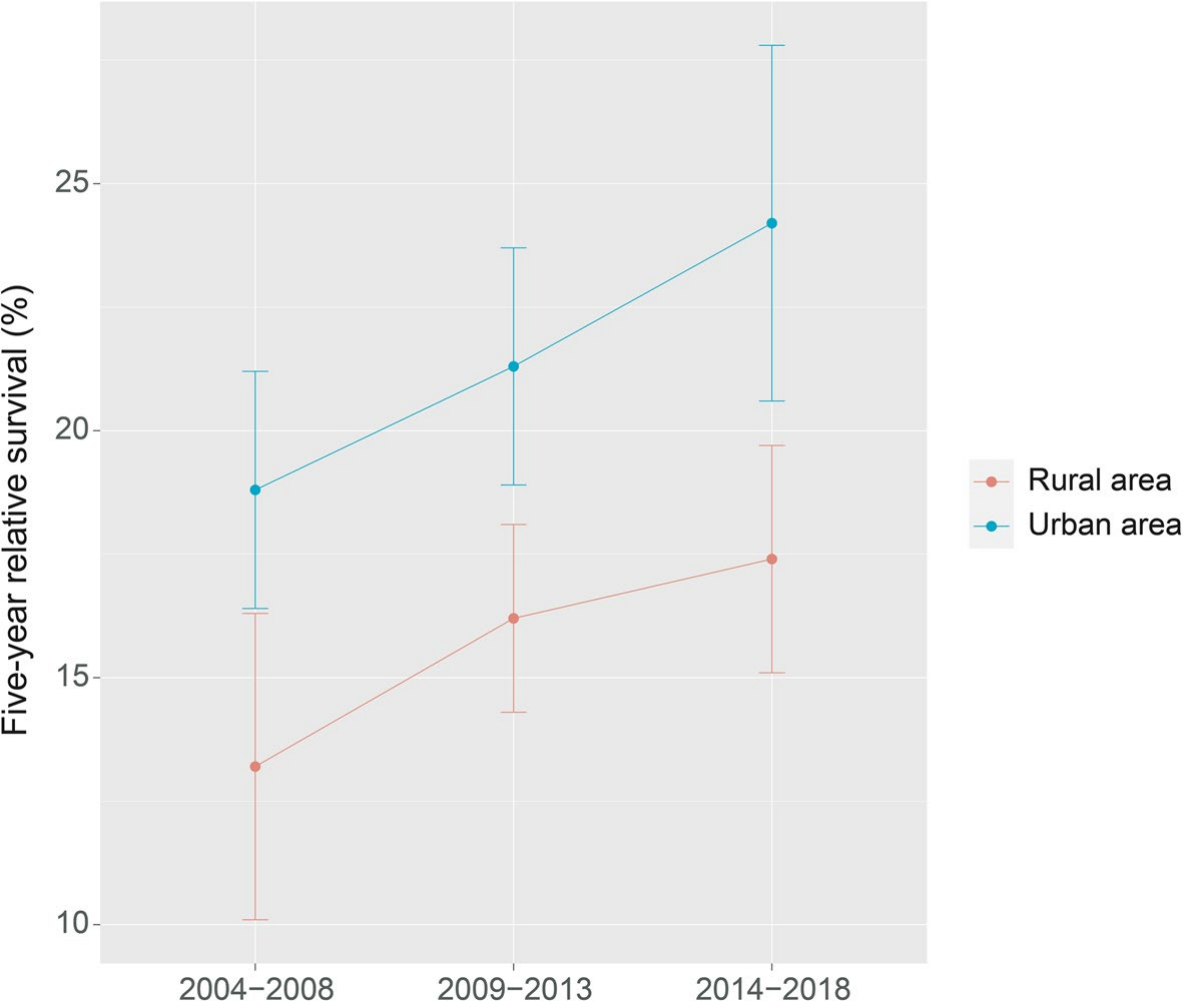
Five-year relative survival of pancreatic cancers for urban and rural areas in 2004–2008, 2009–2013, and 2014–2018

Additionally, the 5-year RS showed an increasing trend over the three periods (2004–2008, 2009–2013, and 2014–2018). Figures 1, 2 and 3 depicts the changes in the survival trend under sex, age at diagnosis, and area stratification, respectively. The estimates of 5-year RS of females were higher than males in all three periods, and the gap between the predicted 5-year RS obtained from male and female patients during 2014–2018 increased compared to 2009–2013 (Fig. 1). Moreover, except for the age group of 65–74 years, the 5-year RS of patients at other diagnosed ages showed an upward trend in all three periods, and the most pronounced improvement was observed among patients 55–64 years of age (Fig. 2). Compared with patients living in urban areas, the difference in the 5-year RS between the two periods (2009–2014 and 2014–2018) was more prominent for rural patients (Fig. 3).

## Discussion

Our study provided the most up-to-date (during 2014–2018) estimates of 5-year RS and survival trends for patients with pancreatic cancer from Taizhou, eastern China, reaching 18.9% overall. While women had higher 5-year RS than men (23.3% versus 14.7%), urban areas had higher 5-year RS than rural areas (24.2 versus 17.4%). We also found a transparent age gradient, declining from 30.3% for age at diagnosis < 45 years to 11.2% for age > 74 years. Additionally, there is a clear increasing trend in 5-year RS during 2004–2018 overall and the stratification by sex, age at diagnosis, and region.

Our overall 5-year RS (18.9%) finding during 2014–2018 was higher than 7.2% for pancreatic cancer during 2012–2015 from 17 population-based cancer registries in China [20]. Nevertheless, our result is plausible due to the following relevant factors. Taizhou of Zhejiang Province is a city with a fast-growing economy and a perfect medical insurance system located in coastal eastern China. The awareness rate of core knowledge about cancer prevention in Zhejiang is relatively high. Therefore, well-educated people in Taizhou attach greater importance to physical health than people from other regions. They may be more willing to take medical examinations, leading to improved rates of early detection and higher rates of surgical resection because of high income, thus increasing survival [22, 23].

Furthermore, more considerable survival improvements were observed in younger patients who were apt to benefit from advances in clinical treatment strategies. A study overviewed cancer survival across seven high-income countries reported increased pancreatic cancer survival, especially for patients younger than 75 years at diagnosis [24]. Dongen et al. also found that patients with pancreatic cancer < 60 years underwent resection despite less stage I disease and had superior overall survival [25]. Besides, the report of 7.2% during 2012–2015 for China was predicted using a hybrid analysis, as the study population was patients diagnosed with cancer between 2003 and 2013, and there is not enough data to use cohort or period approaches for the latest years of follow-up. Existing data could bias the predicted 5-year RS for patients. As a result, the 5-year RS of pancreatic cancer in Taizhou tends to be more optimistic than in other regions. The mentioned reasons contributed to the steadily improved survival as well, compared to no significant improvements for pancreatic cancer from cancer statistics in China.

The 5-year RS of pancreatic cancer patients was further stratified by sex, age at diagnosis, and region. Men had a higher incidence rate and poorer prognosis than women, in accordance with worldwide statistics supporting that male is an unmodifiable risk factor for pancreatic cancer [26, 27]. Some studies investigated the survival difference between sexes for pancreatic cancer, but there was no sufficient statistical evidence despite the sex difference [28, 29]. Lifestyle differences and genetic factors may partially explain the slight male predominance. The average age at diagnosis was 70 years, and age at diagnosis over 60 contains the most significant number of pancreatic cancer patients. The 5-year RS decreased with the increasing age at diagnosis, suggesting that younger pancreatic cancer patients had a higher survival rate, which is consistent with other studies [30, 31]. The differences in survival rates between urban and rural areas can be attributed to several factors, such as the disparity in socioeconomic status, health education, and the unbalanced allocation of medical facilities and resources [32]. As the rural areas are less densely populated regions outside the city center, leading to relatively low level of medical staff and outdated equipment will further aggravate the allocation inefficiency in rural areas compared to urban areas. Additionally, the differences in prevalence and morbidity rates between rural and urban areas could also contribute to differences in survival rates. A study analyzing the incidence and mortality data of cancers in Zhejiang Province, where Taizhou city is located, found higher cancer burden, incidence, and mortality rates in rural areas compared to urban areas [33], suggesting that lack of awareness about cancer prevention among rural residents and limited access to medical resources may be contributing factors.

Moreover, we assessed the survival trend in patients with pancreatic cancer from 2004 to 2018 based on period analysis. We observed an increasing trend in the overall 5-year RS during the study period, implying an improvement in the prognosis of pancreatic cancer patients in Taizhou. The improvement might be related to many reasons. The universal health insurance reform launched in 2009 led to more than 90% of Chinese benefiting from extensive health insurance coverage, reducing the medical burden of citizens to a large extent [34]. Most patients could afford and actively attend medical treatment, improving survival rates. The region’s economy and education advantages are undoubtedly contributing factors as well.

Meanwhile, our understanding of the development and progression of pancreatic cancer has improved substantially. Therefore, targeted- and immune- therapy strategies emerged and benefitted specific molecular subgroups of tumors [35]. However, most pancreatic cancer patients still lack specific efficient therapy strategies. With an increasing understanding of the treatment barriers imposed by the tumor-associated stroma and the development of novel approaches to reengineering the tumor microenvironment, there will be breakthroughs in favor of effective anticancer responses [36, 37].

Despite the instant and accuracy of the period analysis method, it may be too optimistic about applying it if early detection or timely treatment only delays the time to death caused by cancer and does not improve the chances of cure. This study further stratified analysis on clinical-pathological characteristics, including tumor type or TNM classification, was not conducted because of the limited number of cases obtained from only 4 registries and the lack of more detailed information. Therefore, further investigations are needed and encouraged to assess the longer-term survival of pancreatic cancer using cancer registry data with larger size and more specific information.

## Conclusions

In conclusion, the results suggested that the survival of pancreatic cancer patients gradually increased from 2004 to 2018. Our findings provide a scientific basis for formulating cancer prevention, control policies, and public health interventions. Period analysis tended to provide more up-to-date and accurate long-term survival for pancreatic cancer patients using population-based cancer registry data, which may be worthy of further promotion and spread applications.

## Supplementary Information

**Additional file 1.**

## Data Availability

The data used and/or analysed during this study are available from the corresponding author upon reasonable request.
