# Supplementary material for: Use of period analysis to provide a timely assessment of 5-year relative survival for pancreatic cancer patients from Taizhou, eastern China
Source: BMC Cancer. 2023 Jul 10;23:642. doi: 10.1186/s12885-023-11119-3 (PMC10331994; doi:10.1186/s12885-023-11119-3)
Supplement: Supplementary file 1 — Additional file 1. [file 12885_2023_11119_MOESM1_ESM.docx]

**Supplementary**

**Period analysis was used to calculate 5-year RS**

First, we assessed the 5-year relative survival (RS) of patients diagnosed in 2014–2018 by period analysis and divided patients into the newly diagnosed patients during 2014-2018 and those diagnosed from 2009 to 2013 but still alive between 2014-2018. The period analysis uses survival experience observed in a specified calendar period, which included left censoring of observations, and survival observations are right truncated at the end of the calendar period. Additionally, the method calculated the 1-year RS *Si* at the *i* year of follow-up based on collecting a life table from cancer registries. The formula can be written as:

$$\boldsymbol{S}_{\boldsymbol{i}}\mathbf{=1-}\frac{\boldsymbol{d}_{\boldsymbol{i}}}{\boldsymbol{n}_{\boldsymbol{i}}\mathbf{-}{\boldsymbol{c}_{\boldsymbol{i}}}/\mathbf{2}}$$

In this formula, where *n_i_* denoted the population at the beginning of the *i* year of follow-up, *d_i_* denoted the number of deaths at the end of *i* year of follow-up, and *c_i_* denoted the number of censored data in *i* year. The estimate of survival by the end of the follow-up year *k* (*s_k_*) was derived by multiplying the one-year survival rate of the conditions of *k* years. The formula can be written as:

$$\bar{\boldsymbol{S}_{\boldsymbol{k}}}\mathbf{=}\prod_{\boldsymbol{i}\mathbf{=1}}^{\boldsymbol{k}} \boldsymbol{S}_{\boldsymbol{i}}$$

RS was the ratio of the observed survival divided by the expected survival. The formula was as follows:

$$\boldsymbol{R}_{\mathbf{i}}\mathbf{=}\frac{\bar{\boldsymbol{S}_{\boldsymbol{k}}}}{\boldsymbol{S}_{\boldsymbol{k}}^{\mathbf{*}}}$$

Where $\bar{S_{k}}$ denoted observed survival, and$S_{k}^{*}$ denoted the expected survival, which is calculated using the Ederer II method. When *k*=5, the estimates of 5-year RS derived from this formula. According to the Greenwood method, the point estimate of the RS and its standard error were calculated.
